# Primary mitral regurgitation: Toward a better quantification on left ventricular consequences

**DOI:** 10.1002/clc.24190

**Published:** 2023-11-10

**Authors:** Antoine Neveu, Samy Aghezzaf, Emmanuel Oger, Guillaume L'official, Elizabeth Curtis, Elena Galli, David Montaigne, Augustin Coisne, Erwan Donal

**Affiliations:** ^1^ CHU Rennes, Inserm, LTSI – UMR 1099 University of Rennes Rennes France; ^2^ Inserm, CHU Lille, Institut Pasteur de Lille, U1011 ‐ EGID University of Lille Lille France; ^3^ Clinical section Fundamental and Clinical Pharmacology, CHU Rennes University of Rennes Rennes France

**Keywords:** exercise echocardiography, left ventricular global longitudinal strain, myocardial work, primary mitral regurgitation, prognosis

## Abstract

**Background:**

Left ventricular end‐systolic diameter (LVESD) and ejection fraction (LVEF) are the parameters to look for when discussing repair in asymptomatic patients with a primary mitral regurgitation (PMR). Loading conditions are altering LV‐function quantification. LV‐myocardial work (LVMW) is a method based on pressure‐strain loops.

**Hypothesis:**

We sought to evaluate the additive value of the LVMW for predicting clinical events in patients with PMR.

**Methods:**

103 patients (66% men, median age 57 years) with asymptomatic severe PMR were explored at rest and during an exercise stress echocardiography. LV myocardial global work index (GWI), constructive work (GCW), wasted work (GWW), and work efficiency (GWE) were measured with speckle‐tracking echocardiography at rest and low workload. The indication for surgery was based on the heart teams' decision. The median follow‐up was 670 days.

**Results:**

Clinical events occurred for 50 patients (48.5%) with a median of event‐free survival distribution of 289 days. Systolic pulmonary artery pressure (sPAP) at rest was 32.61 ± 8.56 mmHg and did not predict the risk of event like LVEF and LVESD. Changes in, GLS (hazard ratio [HR] 0.55; 95% confidence interval (Cl): 0.36–0.83; *p* = .005), GWI (HR 1.01; 95% Cl: 1.00–1.02; *p* = .002) and GCW (HR 1.85; 95% Cl: 1.28–2.68; *p* = .001) in addition to Left Atrial Volume Index (HR 1.73; 95% CI: 1.28 – 2.33; *p* < 0,001) were independent predictors of events.

**Conclusion:**

Changes in myocardial work indices related to low‐dose exercise are relevant to best predict PMR patient prognosis It might help to better select patient's candidate for “early‐surgery.”

## Graphical abstract

Selecting asymptomatic patients with a primary mitral regurgitation that are at risk, remains a clinical issue. Echocardiography and the advances in the use of the speckle‐tracking tools could help The present work demonstrates that global longitudinal and its use for the calculation of myocardial work indices is a tool of interest. It is demonstrated here that myocardial work could be the best tool we have for assessing the consequences of the mitral regurgitation on the left ventricle.

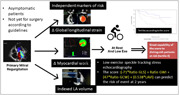

**Abbreviations**
EROA: effective regurgitant orifice area
ExE: exercise echocardiography
GCW: Global Constructive Work
GLS: global longitudinal strain
GWE: global work efficiency
GWI: Global Work Index
GWW: global waste work
LAVI: Left Atrial Volume Index
LV: left ventricle
LVEF: left ventricular ejection fraction
LVESD: left ventricular end‐systolic diameter
MW: myocardial work
PACS: peak atrial constructive strain
PALS: peak atrial longitudinal strain
PMR: primary mitral regurgitation
PSL: pressure strain loop
RV: right ventricle
sPAP: systolic pulmonary artery pressure
TAPSE: tricuspid annular plane systolic excursion

## 1 INTRODUCTION

Primary mitral regurgitation (PMR) is the second most frequent valve disease requiring surgery in western countries.^1^ PMR arises due to degenerative changes of the mitral valve and apparatus and if left unrepaired is association with progressive left ventricular (LV) dysfunction and congestive heart failure. Mitral valve repairs or replacement are the only treatments available for severe MR and are associated with improved prognosis and survival.^2^ Without intervention, symptomatic patients have an annual rate of death of 5% or more.^3^

According to current European Society of Cardiology (ESC)^4^ and American College of Cardiology/American Heart Association (ACC/AHA)^5^ guidelines, mitral surgery is associated with excellent outcomes^6^ in significant PMR when performed before the onset of symptoms, LV dysfunction or dilatation. LV dysfunction and dilatation are defined as an LV ejection fraction (LVEF) < 60% and an LV end‐systolic diameter (LVESD) > 40 mm, respectively.

For asymptomatic patients, the optimal timing of surgery is still debated. There is increasing evidence showing that mitral valve surgery performed before Class I indications are reached confers a long‐term survival advantage.^6^, ^7^, ^8^, ^9^

For decades, LVEF has been the mainstay metric for classifying patients. Nearly all clinical outcome trials have utilized LVEF. As such, clinical guidelines remain anchored to LVEF. Nonetheless, limitations of LVEF have long been recognized, including lack of consistent pathophysiological basis, dependency on hemodynamic loading conditions and rhythm status, insensitivity to subtle changes in contractility, and moderate reproducibility owing to high intra‐observer and interobserver variability^10^, ^11^

LV GLS has recently emerged as a potentially strong method for predicting long‐term prognosis, in both pre and postoperative patients, to its potential to identify early LV dysfunction in patients with preserved LVEF.^12^, ^13^, ^14^, ^15^, ^16^ Also, exercise strain was of benefit to identify patients more likely to develop post‐op LV (testing of the contractile reserve).^14^ LV‐GLS does not integrate afterload,^17^ which represents still a limit even when looking at change from rest to submaximal exercise stress test.^15^

Russel et al.^18^ have developed a new tool, based on LV pressure strain‐loops (LV‐PSL), to assess myocardial work (MW), which explores LV function taking into account afterload. This method has been validated in patients with heart failure and was associated with outcome.^19^ Clinical applications of this global myocardial work index (GWI) and its components (constructive, wasted work, and work efficiency) as well as its prognostic implications, in patients with PMR, have not been evaluated.

Accordingly, the aim of our study was to investigate myocardial work parameters and longitudinal strain and their change with exercise (testing myocardial reserve) in patients with significant PMR and the potential prognostic values in association with known predictors.

## 2 METHODS

### 2.1 Patients' population

We conducted a multicenter retrospective observational study in Rennes and Lille University hospitals. Patients with moderate‐to‐severe PMR were identified from the echocardiographic database. All patients were asymptomatic and were followed on a regular basis as recommended.^4^, ^5^

Patients with inadequate echocardiographic recordings or without blood pressure measurements at the time of the echocardiography were excluded (*N* = 25).

The study population consisted of patients with moderate to severe primary MR, referred to Lille and Rennes University Hospital for risk stratification. Patients underwent exercise echocardiography (ExE) to confirm MR severity and its consequences. ExE was performed according to a standardized protocol including a complete echocardiography at low‐level exercise (as recommended for the “diastolic stress echocardiography”).^20^ This has been done in Mascle et al.^15^ Patients were excluded in case of severe mitral annulus calcification, significant mitral stenosis (mean gradient > 5 mmHg), more than moderate concomitant valvular heart disease at rest, prior cardiac surgery, Class I recommendation for mitral valve surgery, including atrial arrhythmias, limited echocardiographic quality rendering image quality unable to assess strain and LVEF or inability to perform exercise. The local ethics committee approved the protocol, and all patients gave informed consent.

Clinical data collected demographics characteristics, the grade of the MR, the presence of symptoms, New York Heart Association functional class, cardiovascular risk factors, comorbidities, treatments, and biological results of the patients. Follow‐up was ensured on a regular basis at the heart valve Clinics every year.

### 2.2 Echocardiography examination

Patients underwent standard trans‐thoracic echocardiography (Vivid e95, GE Healthcare, with a 4Vc‐D [2409357] transthoracic probe). The exam was done at rest and under exercise echocardiography on a tilt table. All exercise two‐dimensional and Doppler data were obtained at rest and at low load (target heart rate of 110–120 bpm), as recommended for a diastolic stress test and for testing the contractile reserve,^20^ with an echocardiography guided by the symptoms. Echocardiographic parameters were systematically measured at Rennes' and Lille'sechocardiographic Corelab, using EchoPAC R4 software (GE Healthcare). All acquisitions and measurements were performed according to guidelines by an experienced echocardiographer.

The assessment of mitral regurgitation severity was performed according recommendations,^21^, ^22^ using a multiparametric approach including the effective regurgitant orifice area (EROA) and the volume of regurgitation. For the right ventricle (RV), tricuspid annular plane systolic excursion (TAPSE) and S'‐wave velocityof the tricupid annulus were measured from the four‐chambers apical view. Systolic pulmonary artery pressure (sPAP) was estimated according to the Bernoulli equation by measuring maximal tricuspid regurgitant jet velocity, in combination with an estimation of the right atrial pressure.

The LV‐GLS and the AFI‐RV free wall strain were obtained using dedicated software. AFI‐LA software was used for the left atrial (LA) peak reservoir strain (%) (peak atrial longitudinal strain [PALS]) and pump strain (%) (peak atrial constructive strain [PACS]) assessment.

#### 2.2.1 Myocardial work

Myocardial Work (MW) and related indices were estimated as a function of time throughout the cardiac cycle by the combination of LV strain data and a noninvasively estimated LV pressure curve, as previously described by Russel et al.^18^

The estimated LV pressure curve derives from noninvasively acquired brachial artery cuff pressure and is generated by adjusting the profile of a reference LV pressure curve according to the duration of the isovolumic and ejection phases, as defined by timing of aortic and mitral valve events by echocardiography. Peak systolic LV is considered equal to peak arterial pressure. The LV PSL area is constructed from the LV pressure curve combined with the GLS.

**Figure 1:**
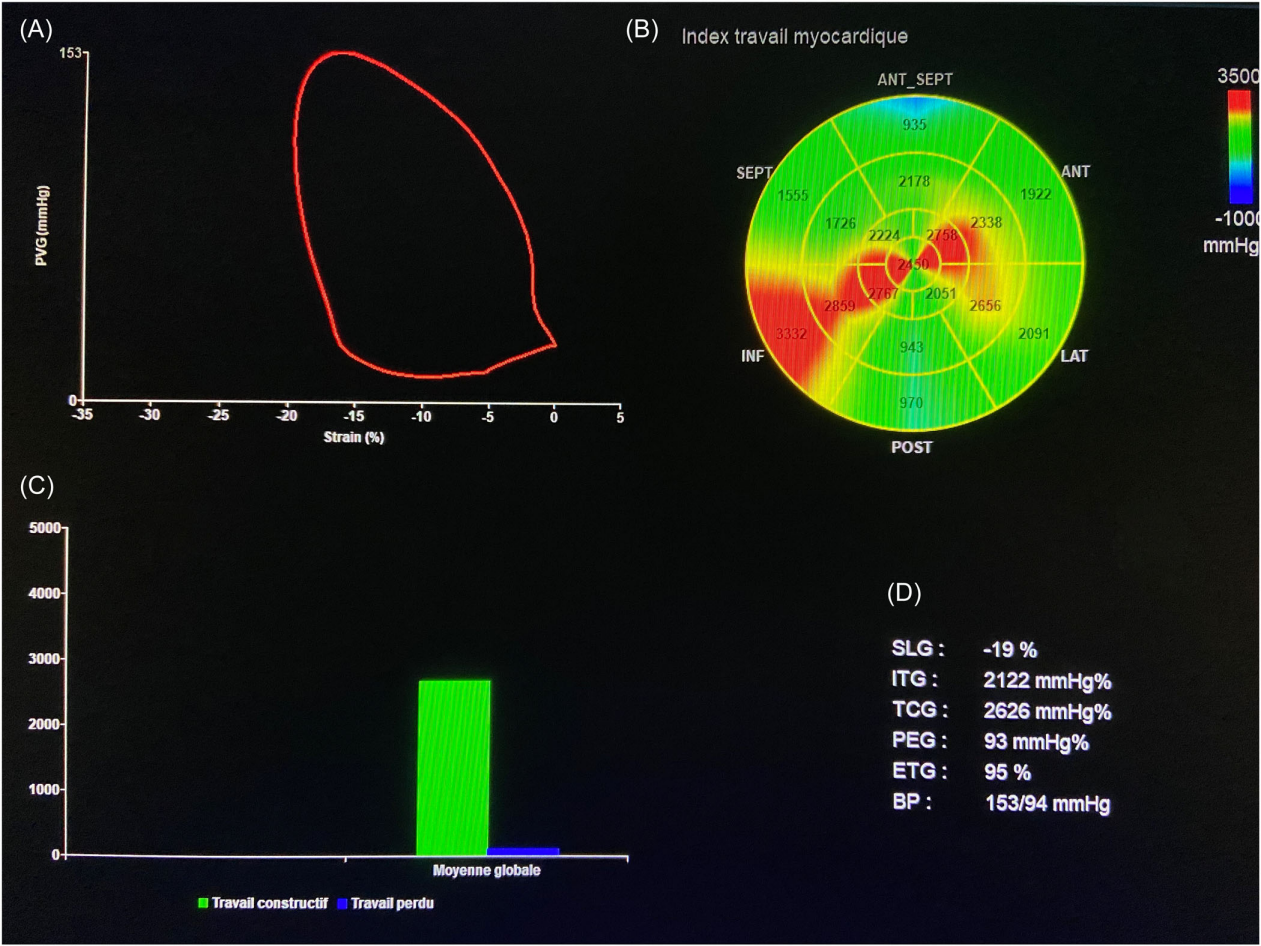
Myocardial work parameters in a patient with primary mitral regurgitation. (A) LV pressure–strain loop; (B) bull's eye of GWI; (C) bar graph representing GCW and GWW; and (D) results from myocardial work analysis. SLG = GLS; ITG = GWI; TCG = GCW; PEG = GWI; ETG = GWE. BP, blood pressure; GCW, global constructive work; GLS, global longitudinal strain; GWI, Global Work Index; GWW, global waste work; LV, left ventricle.

The following parameters were calculated at rest and at low load:

− Global Work Index (GWI) is the total work within the area of the LV PSL, calculated from mitral valve opening to mitral valve closing.
− Global constructive work (GCW) is the work performed by the LV and which is contributing to the LV ejection during the systole. This included the shortening of the myocytes during the systole and the lengthening of the myocytes during isovolumic relaxation.
− Global wasted work (GWW) is the work performed by the LV, which is not contribute to the LV ejection during the systole. This included the lengthening of the myocytes during the systole and the shortening of the myocytes during isovolumic relaxation.
− Global work efficiency (GWE) is the ratio of GCW to the sum of GCW and GWW and expressed as a percentage. It reflects the efficiency of the energy expended through the cardiac cycle.
− Figure 1 displays the main parameters studied.

### 2.3 Outcome

The primary endpoint was the following clinical events recorded based on the regular follow‐up at the heart valve clinics: the onset of symptoms, LV dysfunction or dilatation reaching a Class I surgical indication, and survival at 2 years.

### 2.4 Statistical analysis

The distribution of continuous variables was checked graphically for departure from normality and reported either as mean ± standard deviation or median (25th and 75th percentiles). Categorical variables are reported with frequency and percentage of each attribute. Correlations between echocardiographic parameters were assessed using Pearson or Spearman correlation coefficient along with a graphical approach (matrix plot).

Cox‐proportional hazards regression model was fitted to assess whether relative difference (computed as follows: (exercise value – resting value)/resting value) for GLS and for MW parameters were associated with clinical event onset. Variables with a *p*‐value < .10 on univariate analysis were entered into a multivariable model. For all statistical tests, a *p*‐value < .05 was considered as statistically significant.

We tested functional forms of covariates and proportionality assumptions through graphical and numerical methods based on the cumulative sums of martingale residuals.^23^

We developed a score chart to facilitate the calculation of a patient's two‐year survival by dividing the regression coefficients of the model developed by the lowest value, rounding to the nearest integer to provide a number for each of the predictors; the score was thus the sum of products of predictor values times those numbers. Patients were classified into three groups according to the tertile of the score distribution. A Kaplan–Meier curve was constructed based on these groups. The score is calculated as follows: (−71 × ratio GLS) + ratio GWI + (47 × ratio GCW) + (0.538 × LAVI).

## 3 RESULTS

### 3.1 Description of the patient population

103 patients (median age 57 years, 68 (66%) men) underwent resting and exercise transthoracic echocardiography (56 in Lille and 47 in Rennes). Characteristics of these patients are displayed in Table 1. The anatomic remodeling was more marked for the LA than the LV.

**Table 1:** Clinical and biological characteristics.

Variables	Statistics
Age, years	57 ± 16
Gender, male	68 (66.0)
NYHA	
Class I	100 (97.1)
Class II (atypical symptoms)	3 (2.91)
Hypertension	25 (24.5)
Smoking habit	13 (12.7)
Dyslipidemia	17 (16.7)
Diabetes mellitus	4 (3.92)
Medication	
Beta‐blockers	23 (22.3)
ACEi/ARB	25 (24.5)
Antiplatelet agent	17 (16.7)
Statins	13 (12.7)
NT‐pro‐BNP (ng/mL)	130 [64–246]
Hemoglobin (g/dL)	14.7 [13.9–16.0]
MR severe (IV)	73 (70.9)

*Note*: Values are mean ± standard deviation or median [25th–75th percentiles] or frequency (percentage).

Abbreviations: ACEi, angiotensin‐converting enzyme inhibitor; ARB, angiotensin receptor blocker; MR, mitral regurgitation; NYHA, New York Heart Association.

Seventy‐three patients (70,9%) had severe MR. Almost all patients (97,1%) were in NYHA functional class I. Very few patients with atypical (and very probably non‐related to the mitral valve disease) symptoms were included.

### 3.2 Echocardiographic data at rest and at low exercise

The main echocardiographic parameters at rest are detailed in Table 2A,B. The median effective regurgitant orifice area (EROA) was 38.0 [25.5–47.5] mm^2^. The mean LVEF was normal with 66.44% ± 6.71% as well as the LV GLS (−20.0% ± 3.21%). The median LV end‐systolic diameter (LVESD) was 34.0 [32.0–39.0] mm. For the left atrial, the mean volume index (LAVI) was 53.56 ± 18.80 mm/m^2^, the mean strain PALS was 24.11% ± 7.97%, and the mean strain PACS was 9.21% ± 4.01%. Regarding the right ventricle (RV), the mean TAPSE was 25.06 ± 5.23 mm, the mean tricuspid S way velocity was 14.72 ± 3.07 cm/s, and the RV strain‐free wall was −24.12% ± 5.20%. The mean value of sPAP was normal with 32.61 ± 8.56 mmHg, and the TAPSE/sPAP ratio was 0.82mm/mmHg ± 0.25 mmHg.

**Table 2:** A. Resting echocardiographic parameters.

Variables	Statistics
LA strain PALS (%)	24.11 ± 7.97
PACS (%)	9.21 ± 4.01
RV strain global (%)	−20.53 ± 3.43
RV strain‐free wall (%)	−24.12 ± 5.20
LVEF (%)	66.44 ± 6.71
LVESD (mm)	34.0 [32.0–39.0]
LAVI (ml/m²)	53.56 ± 18.80
EROA (mm²)	38.0 [25.5‐47.5]
Systolic PAP (mm Hg)	32.61 ± 8.56
TAPSE (mm)	25.06 ± 5.23
Tricuspid S way velocity (cm/s)	14.72 ± 3.07
TAPSE/Systolic PAP (mm/mmHg)	0.82 ± 0.25
GLS (%)	−20.0 ± 3.21

B. Low exe echocardiographic parameters	
Variables	Mean ± standard deviation
LVEF (%)	66.53 ± 7.50
Systolic PAP (mmHg)	50.80 ± 12.51
TAPSE (mm)	27.37 ± 5.96
TAPSE/systolic PAP (mm/mmHg)	0.58 ± 0.19
GLS (%)	−20.2 ± 3.09

*Note*: Values are mean ± standard deviation or median [25th–75th percentiles] or frequency (percentage).

Abbreviations: EROA, effective regurgitant orifice area; GLS, global longitudinal strain; LA, left atrial; LVEF, ventricular ejection fraction; LVESD, left ventricular end‐systolic diameter; LAVI, left atrial volume index; PACS, peak atrial contraction strain; PALS, peak atrial longitudinal strain; PAP, systolic pulmonary artery pressure; RV, right ventricle; TAPSE, tricuspid annular plane excursion.

Table 2 displays the main echocardiographic parameters at low Exe. The mean LVEF was 66.53% ± 7.50% and the GLS was −20.2% ± 3.09%. The mean TAPSE was 27.37 ± 5.96 mm. The mean value of sPAP was 50.8 ± 12.51 mmHg, and the TAPSE/sPAP ratio was 0.58 ± 0.19 mmHg.

### 3.3 Description of myocardial work parameters at rest and at low exercise

The myocardial work parameters at rest and at low exe are described in Table A3 bis. At rest, the mean GWI was 2039 mmHg% [2374–2687], the mean GCW was 2621 mmHg% ± 570, the mean GWW was 137 mmHg% [101–208], and the mean GWE was 93% [91–96]. At low Exe, the mean GWI was 2309 mmHg% ± 546, the mean GCW was 3062 mmHg% ± 609, the mean GWW was 203 mmHg% [151–323] and the mean GWE was 90.9% ± 4.48.

### 3.4 Survival analysis

The median follow‐up was 670 days. In our study, clinical events occurred in 50 patients (48.5%) with a median of event‐free survival distribution of 289 days.

On univariate Cox regression analysis, only GWI (relative difference 0.12 [−0.04; 0.27], *β* = .045, *p* = .003) was significantly associated with clinical event onset (Table A3 bis).

After multivariate adjustment, using Cox regression analysis with a selection of variables with a *p*‐value < .10 on univariate analysis, GLS (hazard ratio [HR] 0.58; 95% confidence interval [Cl]: 0.38–0.88; *p* = .01), GWI (HR 1.01; 95% Cl: 1.00–1.02; *p* = .001), GCW (HR 2.05; 95% Cl: 1.38–3.03; *p* < .001) and LAVI (HR 1.73; 95% CI: 1.28–2.33; *p* < .001) were independent predictors of clinical events (Table 3). In our study, LVEF and LVESD were not significantly associated with clinical event onset (*p* > .05).

**Table 3:** Univariate and multivariate estimates for the association between relative differences for global longitudinal strain, myocardial work, and other echocardiographic parameters and clinical event onset (50 events out of 103 observations).

			Univariate	Multivariate*	
	Missing/events	Unit	HR (95% CI)	HR (95% CI)	*p*‐Value
GLS	7/47	.15	0.72 (0.51–1.02)	0.58 (0.38–0.88)	.0105
GWI	16/44	.30	1.01 (1.00–1.02)	1.01 (1.00–1.02)	.0011
GCW	16/44	.30	1.31 (0.95–1.76)	2.05 (1.38–3.03)	.0003
GWW	16/44	1.40	0.88 (0.65–1.19)		
GWE	16/44	.05	1.11 (0.81–1.54)		
LVEF	0/50	7	0.99 (0.73–1.33)		
LVESD	0/50	10	1.52 (0.99–2.33)	1.23 (0.80–1.90)	
LAVI	0/50	20	1.73 (1.32–2.27)	1.73 (1.28–2.33)	.0003
EROA	15/47	20	1.61 (1.19–2.18)	1.30 (0.92–1.83)	
Systolic PAP	4/47	.25	1.00 (0.99–1.01)		

*Note*: Relative difference was computed as follows: (exercise value − resting value)/resting value.

Abbreviations: CI, confidence interval; EROA, effective regurgitant orifice area; GCW, global constructive work; GLS, global longitudinal strain; GWI, Global Work Index; GWW, global waste work; HR, hazard ratio; LVEF, ventricular ejection fraction; LVESD, left ventricular end‐systolic diameter; LAVI, left atrial volume index; PAP, systolic pulmonary artery pressure.

* Missing/events: 16/44.

**Figure 2:**
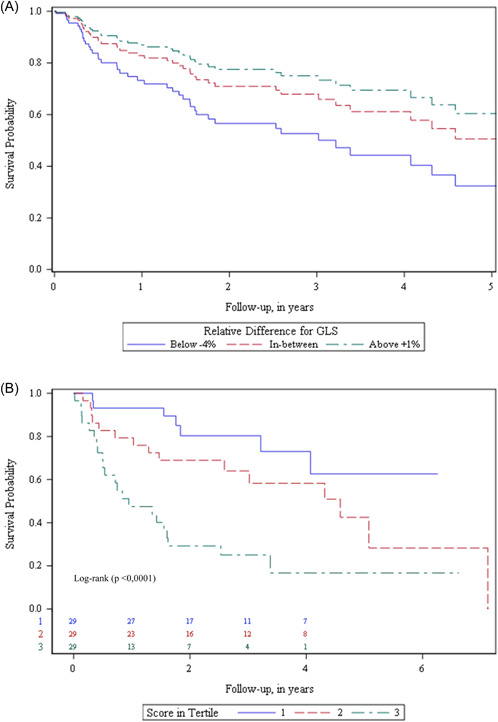
(A) Event‐free survival plot for a set of covariates: three‐level‐level (using first and third quartiles of the distribution) for the GLS variables and the average values for GWI, GCW, and LAVI. (B) Better Event‐free survival plot according to tertiles of the score combining GLS, GWI, GCW, and LAVI. GCW, global constructive work; GLS, global longitudinal strain; GWI, Global Work Index; GWW, global waste work; LAVI, left atrial volume index.

Figure 2A,B is providing Kaplan–Meier curves for GCW, GWI, and LAVI according to quartiles of distribution. Patients with a relative difference in GLS of more than −4% had the worst event‐free survival probability.

We developed a score chart to facilitate the calculation of a patient's 2‐year, combining GLS, GCW, GWI, and LAVI. Table A1 bis (Appendix) gives the score and distribution in tertiles. Table A2 bis (Appendix) and Figure 2A,B displays the patient's 2 years of event‐free survival, according to the tertiles of the score. At 2 years, event‐free survival probability of the first tertile (score 0–32) was 0.80 (95% Cl (0.58–0.91); *p* < .0001). For the second tertile (score 32–44) it was 0.69 (95% Cl (0.49–0.82); *p* < .0001), and for the third tertile (score 44–111), it was 0.29 (95% Cl (0.14–0.46); *p* < .0001).

## 4 DISCUSSION

In the present study of asymptomatic patients with moderate‐to‐severe primary mitral regurgitation, normal LV dimensions, and preserved LVEF, who underwent rest and submaximal exercise echocardiography, we found that altered baseline resting LV‐GLS and change in GLS but also change in Myocardial Work parameters were independently associated with the primary end‐point (onset of symptoms, LV dysfunction or dilatation leading to surgical indication), providing additive prognostic value that may help to further identify patients who may benefit from early surgery.

### 4.1 Value of global longitudinal strain

In asymptomatic patients with preserved LVEF, there is a need to identify more sensitive markers of regional myocardial dysfunction. LV‐GLS has helped in demonstrating that subtle LV dysfunction may occur in patients before any drop in ejection fraction and before any LV enlargement. Also, LV biopsy findings in asymptomatic patients with chronic severe MR and normal ejection fraction at the time of MV surgery demonstrate more myofibrillar degeneration, higher xanthine oxidase, and lipofuscin deposition, indicating more oxidative stress compared with normal subjects.^23^ Similar findings have been made by late gadolinium enhancement and cardiac magnetic resonance imaging.^24^, ^25^ Impaired LV‐GLS in asymptomatic patients with severe MR and preserved ejection fraction has been consistently shown to predict postoperative LV dysfunction after MV surgery.^15^, ^26^, ^27^ Witkowski et al.^26^ reported a cut‐off value of LV‐GLS > −19,9% as an independent predictor of long‐term postoperative LV dysfunction.

A previous study reported resting and stress LV‐GLS and found a strong correlation of GLS indexed to LV end‐systolic dimension with postoperative ejection fraction^14^; however, no survival analysis was reported. In our study, a decrease in relative difference of GLS more than −4% is associated with reaching a Class I indication for surgery, which further adds to the evidence that GLS could be utilized as an early marker of LV dysfunction to identify patients who require surgery in PMR. Factors like LV dimensions, LV ejection fraction, or mitral EROA were not significantly associated with prognosis in our study, likely because of a homogeneous study population with an asymptomatic primary MR with normal LVESD and preserved LV ejection fraction.

### 4.2 Myocardial work indices

Myocardial work is a novel and complementary method to the LV‐GLS in the evaluation of LV systolic function. In contrast with LV‐GLS, the MW takes into account the afterload due to a noninvasive estimation of intraventricular pressure during the cardiac cycle.

LV‐GLS measurements and LV‐myocardial work indices (GCW and GWI) were of great value at rest and for testing for the “contractile reserve” during an exercise stress echocardiography. Of note, none of the patients have any documented obstructive coronary artery disease, left‐sided valvular stenosis, or bundle branch block. The wasted work and its changes with exercise, (measuring the “contraction” that does not contribute to the output) was not an independent prognostic marker in our population. The definition of what constitutes “normal” LV‐GLS values in severe MR remains controversial because of the unknown effect of the loading conditions on the GLS.

The mean value of LV‐GLS in the current study (−20.0% ± 3.21) was significantly higher than what was reported in a study of healthy individuals free of cardiovascular disease.^28^

The improved approach for quantifying LV‐systolic function using the myocardial work indices is demonstrated here, at least for the ones quantifying the LV function that is contributing to the output. The values obtained in MR patients are similar as compared to the “normal” values but the delta related to a submaximal exercise is demonstrated valuable.^28^, ^29^

### 4.3 Contribution of exercise echocardiography in combination with longitudinal deformation parameters

As shown in the present study, the difference between rest and submaximal effort of GLS and MW was the best tool for event prediction in this population with asymptomatic severe primary mitral regurgitation. The individual measurement made during exercise did not perform better than the rest measurement. The change, the delta is demonstrated as valuable like it has been shown by Magne et al.^30^ for GLS previously. It is not widely accepted but the concept is already demonstrated.^30^ The used exercise is submaximal; it tests the contractile reserve and also, feasibility is great and image quality remains preserved. The main issue when exercising became the obtention of a blood pressure at the right period of time. The added value for myocardial work indices to GLS is a new reason for pushing forward the stress echocardiography and its combination with speckle‐tracking analyses. The contractile reserve has already been advocated as an important parameter to review, and MW approach allows deeper assessment in that direction. Of course, our first results are pilot, and further confirmations is required but “heart valve teams” should not forget about the value of exercise echocardiography in the decision‐making process for a patient with PMR.

It is not precisely identified in the key tables of recent guidelines, but evidence exists supporting its use.^29^, ^30^, ^31^, ^32^ Recently, Coisne et al.^32^ showed that in patients with ⩾ moderate PMR and discordant symptoms, 25 W exercise pulmonary hypertension and poor aerobic capacity during cardiopulmonary exercise testing are independently associated with adverse events.

### 4.4 New perspectives

Current European and American recommendations^4^, ^5^ are based on LVEF analysis in the Simpson biplane technique and LV measurements to determine whether asymptomatic patients should undergo mitral valve surgery. It is interesting that our patient cohort with asymptomatic PMR has normal LV diameter with LVEDS of 34 mm [32–39] but has dilated LA at 53.56 mL/m^2^ ± 18.80. This is something we observe from time to time, especially in women. Of note, it has been published by others, and LA dilatation in sinus rhythm is by itself an indication IIa for mitral valve repair according to guidelines.^4^

### 4.5 Multiparametric approach

A multi‐parametric approach, including GLS with myocardial work changes obtained via echocardiography might be useful. This would guide the management of this population with asymptomatic primary mitral regurgitation, normal LV dimensions, and preserved LVEF. The score proposed in this study is a potentially complementary tool for decision‐making. Close monitoring seems appropriate when the score is low, whereas a high score might indicate that the patient may benefit from earlier surgical intervention. Specific studies addressing the added value of this score should be performed to include it in daily practice. The graphical abstract image displays the parameters included in the score.

### 4.6 Limitations

This study has a few limitations. First, this was an observational retrospective study from two tertiary centers with its inherent selection bias. Second, the number of patients included remains limited, and only a derivation cohort was available for the score. Third, we only included asymptomatic patients with moderate to severe primary MR who subsequently underwent rest and exercise echocardiography. The study population was homogeneous concerning the degree of MR, and hence, mitral EROA was likely not a significant predictor of exercise capacity. Patients who have been symptomatic at baseline did not have the test and are not included in our study. In the future it may be useful to test the “contractile reserve” assessed by myocardial work indices in severe patients in whom uncertainties exist about optimal management strategies.

## 5 CONCLUSION

The variation of myocardial work indices during exercise echocardiography might be relevant when combined to indexed LA‐volume and delta of change in GLS with exercise to predict the occurrence of events in asymptomatic patients with moderate‐to‐severe primary mitral regurgitation. A multi‐parametric score based on these deltas in exercise echocardiography might be useful to improve the management of patients with primary mitral regurgitation.

## CONFLICT OF INTEREST STATEMENT

The authors declare no conflict of interest

## Data Availability

The data underlying this article will be shared on reasonable request to the corresponding author. The data that support the findings of this study are available on request from the corresponding author. The data are not publicly available due to privacy or ethical restrictions.

## 1

Tables A1–A3

**Table A1:** bis distribution of the score (87 observations).

Tertile	*N* Obs	*N* Miss	Minimum	Maximum
1	29	0	0	32
2	29	0	32	44
3	29	0	45	111

**Table A2:** bis: Event‐free survival chart at 2 years according to tertiles of the score combining GLS, GWI, GCW, and LAVI (87 observations).

Tertile	Survival	95% CI
1	0.80	0.58–0.91
2	0.69	0.49–0.82
3	0.29	0.14–0.46

Abbreviations: CI, confidence interval; GCW, global constructive work; GLS, global longitudinal strain; GWI, Global Work Index; LAVI, left atrial volume index.

**Table A3:** bis. Resting and exercise echocardiographic parameters and association of relative difference with clinical event onset.

	Missing	Resting	Missing	Exercise	Relative difference	*β* ± SE	*p*‐Value
GLS (%)	‐	−20.0 ± 3.21	7	−20.2 ± 3.09	0.01 ± 0.14	−2.177 ± 1.180	.065
GWI (mmHg%)	4	2039 [2374‐2687]	16	2309 ± 546	0.12 [−0.04; 0.27]	0.045 ± 0.015	.003
GCW (mmHg%)	4	2621 ± 570	16	3062 ± 609	0.16 [0.05; 0.32]	0.908 ± 0.496	.067
GWW (mmHg%)	4	137 [101–208]	16	203 [151–323]	0.50 [−0.01; 1.42]	−0.088 ± 0.109	.418
GWE (%)	4	93 [91–96]	16	90.9 ± 4.48	−0.01 [−0.04; 0.01]	2.179 ± 3.266	.505

*Note*: Values are mean ± standard deviation or median [25th–75th percentiles]. *β*  ±  SE stands for parameter estimate (Cox regression) and standard error. Relative difference was computed as follows: (exercise value − resting value)/resting value.

Abbreviations: GCW, global constructive work (mmHg%); GLS, global longitudinal strain (%); GWE, global work efficiency (%); GWI, global work index (mmHg%); GWW, global wasted work (mmHg%).
